# Integrative analysis reveals a lineage-specific circular RNA landscape for adipo-osteogenesis of human mesenchymal stem cells

**DOI:** 10.1186/s13287-022-02792-5

**Published:** 2022-03-12

**Authors:** Hai-Bo Huang, Hai-Tao Luo, Na-Na Wei, Miao-Ling Liu, Fei He, Wei Yang, Jun Dong, Xiao-Fei Yang, Fu-Rong Li

**Affiliations:** 1 Translational Medicine Collaborative Innovation Center, Shenzhen People’s Hospital (The Second Clinical Medical College, Jinan University; The First Affiliated Hospital, Southern University of Science and Technology), ShenzhenGuangdong, 518020 China; 2 Guangdong Engineering Technology Research Center of Stem Cell and Cell Therapy, Shenzhen Key Laboratory of Stem Cell Research and Clinical Transformation, Shenzhen Immune Cell Therapy Public Service Platform, Shenzhen, 518020 Guangdong China; 3 Integrated Chinese and Western Medicine Postdoctoral Research Station, Jinan University, Guangzhou, 510632 Guangdong China; 4 Department of Pathophysiology, Key Laboratory of the State Administration of Traditional Chinese Medicine, Jinan University, Guangzhou, 510632 Guangdong China; 5 Agricultural Genomics Institute at Shenzhen, Chinese Academy of Agricultural Sciences, Shenzhen, 518124 China; 6 Kunpeng Institute of Modern Agriculture at Foshan, Foshan, 528200 China

**Keywords:** Mesenchymal stem cells, Osteogenesis, Adipogenesis, Circular RNAs, CRLF1

## Abstract

**Background:**

The balance between osteogenesis and adipogenesis of mesenchymal stem cells (MSCs) is critical to skeletal development and diseases. As a research hotspot, circular RNAs (circRNAs) have expanded our understanding of a hidden layer of the transcriptome. Yet, their roles during adipo-osteogenesis remain poorly described.

**Methods:**

The identity of human MSCs derived from bone marrow and adipose were first determined by flow cytometry, cellular staining, and quantitative polymerase chain reaction (qPCR). Multi-strategic RNA-sequencing was performed using Poly A, RiboMinus and RiboMinus/RNase R methods. Integrative analysis was performed to identify lineage-specific expressed circRNAs. The structural and expressional characteristics were identified by Sanger sequencing and qPCR, respectively. The regulatory effects of adipogenesis-specific circ-CRLF1 were confirmed using siRNA transcfection and qPCR.

**Results:**

We generated a whole transcriptome map during adipo-osteogenesis based on 10 Poly A, 20 RiboMinus and 20 RiboMinus/ RNase R datasets. A total of 31,326 circRNAs were identified and quantified from ~ 3.4 billion paired-end reads. Furthermore, the integrative analysis revealed that 1166 circRNA genes exhibited strong lineage-specific expression patterns. Their host genes were enriched in distinct biological functions, such as cell adhesion, cytokine signaling, and cell division. We randomly selected and validated the back-spliced junction sites and expression patterns of 12 lineage-specific circRNAs. Functional analysis indicated that circ-CRLF1 negatively regulated adipogenesis.

**Conclusions:**

Our integrative analysis reveals an accurate and generally applicable lineage-specific circRNA landscape for adipo-osteogenesis of MSCs and provides a potential therapeutic target, circ-CRLF1, for the treatment of skeleton-related disease.

**Supplementary Information:**

The online version contains supplementary material available at 10.1186/s13287-022-02792-5.

## Background

Mesenchymal stem cells (MSCs) are multipotent clonogenic cells that existed in diverse tissues, including bone, fat, and muscle. Due to the potentials to differentiate into stromal cells and their immunoregulatory properties, MSCs are increasingly recognized as crucial contributors to tissue homeostasis and ideal candidates for cell-based therapies [1]. The normal bone microenvironment requires the balance of osteogenesis and adipogenesis of MSCs, the imbalance of which has been linked to pathophysiologic processes of bone such as aging, obesity, osteopenia, and osteoporosis [2–4]. Therefore, clarifying the adipo-osteogenesis of MSCs will facilitate the application of MSC in regenerative medicine, especially for bone-related diseases.

Circular RNAs (circRNAs) are a novel and large family of non-coding RNAs (ncRNAs) with covalent loop structures formed by head-to-tail splicing [5, 6]. CircRNAs have long been considered to be by-products of transcription due to their inability to be translate into proteins and aberrant splicing events [7]. However, accumulating evidence indicates that circRNAs are involved in the development and pathogenesis inmultiple tissues, including bone [7, 8], by functioning as an inhibitor of microRNA or proteins [6, 9], as structural components of protein complexes [10], or as signaling molecules to recruit proteins [11]. For example, circRNA CDR1as could compete for miR-7-5p, which controls the expression of *WNT5B* by targeting its 3′-untraslated region (3’UTR), to promote adipogenesis and suppress osteogenesis of bone marrow-derived MSCs (BMSCs) [12]. Similarly, hsa_circ_0074834 sponges miR-942-5p, which could target the mRNA of *ZEB1* and *VEGF*, to promote the osteogenesis and angiogenesis of BMSCs; circRNA-vgll3 could regulate miR-326-5p/Itga5 axis to promote osteogenesis of adipose-derived MSCs (AMSCs) [13, 14]. CircFUT10 and hsa_circH19 could target *let-7c* and *PTBP1* proteins, respectively, to control adipogenesis [15, 16].

Although the roles of circRNAs in the lineage determination of MSCs have been becoming more evident, only a few publications have reported the expressions with potential functions of circRNAs at a genome-wide level during adipo-osteogenesis in humans. For instance, the circRNA expression levles during osteogenesis have been reported in BMSCs using microarray or RNase R-treated RNA-sequencing (RNA-seq) [17, 18], and in AMSCs and stem cells from the periodontal ligament and apical papilla using RiboMinus-treated RNA-seq [14, 19, 20]. Adipogenesis-related circRNAs have been reported in human visceral and subcutaneous fat tissue and mouse pre-adipocytes using RiboMinus-treated RNA-seq [21]. However, due to their low abundance compared with linear RNAs, a limited number of circRNAs were detected by only removing ribosomal RNAs during RiboMinus-treated RNA-seq [22], and a biased estimation of circRNA levles may be generated during RNase R treatment and multiple purification steps during RNase R-treated RNA-seq [23]. Integrative analysis of multi-strategic RNA-seq data using appropriate algorithms could improve the accuracy of circRNA identification and quantification with a significantly reduced false discovery rate [24].

In the present study, we produced 50 high-quality RNA-seq datasets, using RiboMinus, Poly A, and RiboMinus/RNase R methods, at five time points during adipo-osteogenesis of human BMSCs and AMSCs. Our integrative analysis identified 31,326 circRNAs shared by MSCs from different tissues, 1,166 host genes of which displayed strong lineage-specific expression patterns. Twelve circRNAs were further validated by both quantitative polymerase chain reaction (qPCR) and Sanger sequencing. Moreover, we found that levels of the circRNA, circ-CRLF1, which is derived from the *CRLF1* locus, were markedly increased during adipogenesis, but not osteogenesis. Moreover, the knock-down of circ-CRLF1 significantly promoted adipogenesis. These datasets will be useful for further investigation of the roles of circRNAs during bone development and dysfunction.

## Materials and methods

### Cell culture

Human bone marrow and adipose-derived MSCs were purchased from American Type Culture Collection (Catalog PCS-500-012, PCS-500-011, ATCC, MD, USA). They were cultured in MesenCult-ACF Plus Medium (catalog number 05448, STEMCELL Technologies, Vancouver, Canada) supplemented with 1% penicillin–streptomycin. They were cultured at 37 ℃ with 5% CO_2_. The cells were passaged at a seeding density of 4 × 10^3^ viable cells/cm^2^ when reaching 80% confluence using Animal Component-Free Cell Dissociation Kit (catalog number 05426, STEMCELL Technologies, Vancouver, Canada).

### Osteogenic and adipogenic differentiation

MSCs were orientally induced under passage 8 when they reached 80% confluence. For osteogenic induction, the cells were washed with 1 × dPBS and then cultured with OsteoMAX-XF Differentiation Medium (catalog number SCM121, EMD Millipore, MD, USA) supplemented with 1% penicillin–streptomycin. The osteogenic medium was half-changed on day 2, 4, 7, 9, and 11, according to the previous study [25]. MesenCult Adipogenic Differentiation Kit (catalog number 05412, STEMCELL Technologies, Vancouver, Canada) supplemented with 1% penicillin–streptomycin was used for adipogenic induction, and all the culture medium was replaced at the same time point mentioned above. The samples were collected on day 0, 7, and 14 for the experiments.

### Flow cytometry

The flow cytometry was performed with antibodies for positive markers: anti-CD73 FITC with related isotype (Catalog Number 561254, 554679, BD Pharmingen, CA, USA), anti-CD105 APC with related isotype (catalog number FAB10971A, IC002A, R&D systems, MN, USA), anti-CD166 PE with related isotype (Catalog Number 343904, 400111, BioLegend, CA, USA); and negative markers: anti-CD14 PE with related isotype (Catalog Number 555398, 555574, BD Pharmingen, CA, USA), anti-CD34 APC with related isotype (Catalog Number 555824, 555751, BD Pharmingen, CA, USA), anti-CD45 APC with related isotype (Catalog Number 555485, 555751, BD Pharmingen, CA, USA). The MSCs at passage 8 were digested into single-cell suspension and washed with 1 × dPBS, and then incubate with those fluorescent-conjugated antibodies for one hour at room temperature. After washing with 1 × dPBS, the cells were detected by flow cytometer (Beckman Coulter DxFLEX, CA, USA) with at least 10,000 events. Data were analyzed by FlowJo (TreeStar, OR, USA).

### Alizarin Red S and oil Red O staining

The cells were fixed in 4% paraformaldehyde for 20 min at room temperature and then washed twice with 1 × dPBS. To detect the matrix mineralization during osteogenesis, the cells were stained with 1% Alizarin Red S (pH 4.2; catalog number G1452, Solarbio, Beijing, China) for 15 min. To visualize the intracellular lipid deposits, the cells were incubated with 60% isopropanol for 5 min, then stained with Oil Red O solution (catalog number G1262, Solarbio, Beijing, China) for 15 min. The stained cells were examined by light microscope (EVOS XL Core, 10 × magnification, Life Technology, PA, USA).

### Immunofluorescence assay

Cells grown in 6-well plates were washed 3 times with PBS, and fixed for 15 min in 4% paraformaldehyde at room temperature and treated with 0.1% Triton X-100 for 15 min and then blocked for 60 min with 5% goat serum in PBS. After that, cells were incubated with rabbit anti-RUXN2 (catalog number 12556, Cell Signaling Technology, MA, USA; dilution 1:800) or rabbit anti-CEBPA (catalog number 8178, Cell Signaling Technology, MA, USA; dilution 1:200) primary antibody at 4 °C overnight and secondary antibodies (Alexa Fluor 488, catalog number 4412, Cell Signaling Technology, MA, USA; dilution 1:500) for one h at room temperature, followed by washing and staining with DAPI (2 μg/ml, catalog number D8417, Sigma-Aldrich, MO, USA) for 5 min. Then stained cells were washed two times with PBS, and images were taken using a Leica microscope (Leica, DMi8, Wetzlar, Germany) with its software LAS X. The average optical density (AOD) of immunoreactivity were normalized by AOD of DAPI in the same field using Image J.

### RNA isolation and quantitative PCR

Total RNA was isolated using TRIzol (Catalog Number 15596026, Invitrogen, CA, USA) and RNeasy Mini Kit (Catalog Number 74104, QIAGEN, CA, USA) according to manufacturers’ instructions. For RNase R treatment, 2 μg of total RNA were incubated with or without 3 U/μg RNase R (Catalog Number RNR07250, Epicentre Technologies, WI, USA) for 20 min at 37 ℃, for 5 min at 70 ℃. Subsequently, complementary DNA was produced using the PrimeScript RT Reagent Kit with gDNA Eraser (catalog number RR047A, Takara, Shiga, Japan), and quantitative real-time reverse transcription polymerase chain reaction (qPCR) was performed using the Real-Time PCR detection system (Thermo Scientific SteponePlus, MA, USA) with TB Green Premix Ex Taq™ II kit (catalog number RR820A, Takara, Shiga, Japan). Relative mRNA expression was calculated using the delta threshold cycle (ΔΔCT) method and normalized to GADPH expression [26]. The PCR primers are listed in Additional file 7: Table S6. In addition, the PCR products for circRNAs were performed with sanger sequencing to validate the back-spliced junction sequences.

### Multi-strategic RNA-seq

For RiboMinus-treated RNA-seq, the ribosomal RNAs were removed by Epicentre Ribo-zero rRNA Removal Kit (Epicentre, WI, USA). For Poly A-treated RNA-seq, the poly-T oligo-attached beads were used to purify poly A-containing RNAs. For RiboMinus/RNase R RNA-seq, the rRNA-depleted RNAs were digested with RNase R (Epicentre, WI, USA) to remove linear RNAs. Whereafter, the strand-specific libraries were performed with the treated RNAs using the rRNA-depleted RNA by NEBNext Ultra Directional RNA Library Prep Kit for Illumina (NEB, MA, USA) following the manufacturer’s recommendations. The sequencing was carried out using the Illumina HiSeq X Ten instrument by the commercial service of Novogene Co., Ltd. (Tianjin, China).

### Bioinformatic analysis

Low-quality sequencing reads and the reads with adapters or with > 10% of ploy-N bases were filtered out. After filtering, the clean reads were used to calculate gene expression values and identify circular RNAs. The read count and TPM (transcripts per million) values of each type of gene were determined using the fast pseudoaligner Kallisto (v0.46.0) [27], which pseudoaligned reads to reference gene models (GENCODE v35) [28]. Then, the read counts and TPMs at the transcript level were summarized to the gene level. Differentially expressed genes (DEGs) were identified using the DESeq2 method [29]. DEGs with fold-change more than two and adjusted *P* value less than 0.01 were retained and used in the following analysis. Gene Ontology (GO) enrichment analysis was performed using DAVID bioinformatics resources 6.8 [30]. According to RNA-seq data and reference gene models, circRNAs were identified using CIRI2 [31]. The expression levels of circRNAs were estimated by CIRIquant with RNase R effect correction model based on RNase R treated and untreated samples [24]. CircAltas 2.0 database was used as the source of the reference circular RNAs [32].

### Oligonucleotides transfection

A predesigned siRNA targeting human circ-CRLF1 (si-circRNA) and negative control (scramble siRNA, si-scramble) were purchased from GenePharma Co., Ltd. (Shanghai, China). The sequences used are listed in Additional file 7: Table S6. MSCs were transfected with siRNA at a final concentration of 20 nM on day 0, 2 and 4 during adipogenesis, using Lipofectamine RNAiMAX (Invitrogen, Cat.No. 13778075), according to the manufacturer’s protocol. Cells were harvested on day 7 post-transfection.

### Statistical analysis

Prism software 5.01 (GraphPad Software, Inc., San Diego, USA) were used to for statistical analysis. A two-tailed Student’s *t*-test was used to compare mean values between two groups. The statistical significance in the comparison of multiple sample sets versus control was performed with Bonferroni’s multiple comparisons test after one-way ANOVA test.

## Results

### Adipo-osteogenic characteristics of inducted human BMSCs

**Fig. 1:**
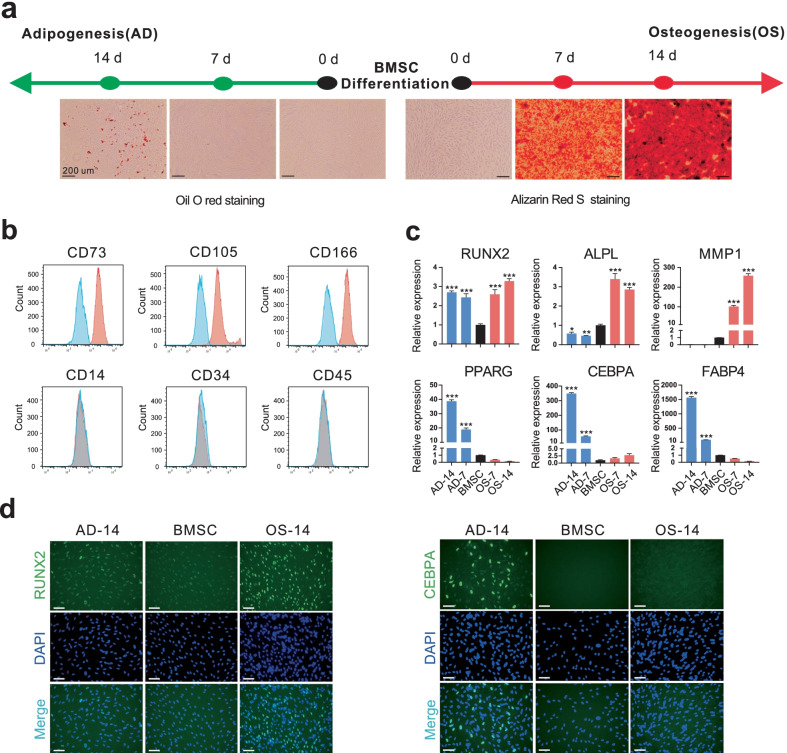
Adipogenesis and osteogenesis of human BMSCs. **a** Oil Red O and Alizarin Red S staining for adipogenic and osteogenic differentiated BMSCs, respectively, on day 0, 7 and 14. **b** Distribution of surface markers of BMSCs. **c** Expression of marker genes. **d** Immunofluorescence staining of marker proteins. DAPI were used to indicate nucleus. Statistically significant differences of genes between multiple time points versus day 0 were performed with Bonferroni’s multiple comparisons test after one-way ANOVA test. Scale bars = 200 μm in pannel **a**. Scale bars = 75 μm in pannel **d**. All data are presented as means ± SD (*n* = 3). *P < 0.05, **P < 0.01, ***P < 0.001

Human BMSCs were expanded and induced using osteogenic or adipogenic medium for 14 days to establish an adipo-osteogenesis system. The expanded BMSCs maintained the typical expression pattern of their surface markers: high CD73, CD105 and CD166 expression levels, and low CD14, CD34 and CD45 expression levels (Fig. 1b; Additional file 1: Fig. S1a). Histological staining indicated the accumulation of extracellular matrix mineralization during osteogenesis and the accumulation of intracellular lipid droplets during adipogenesis (Fig. 1a). Expression levels of osteogenic marker genes, including *RUNX2*, *ALPL*, and *MMP1*, and adipogenic marker genes, including *PPARG*, *CEBPA*, and *FABP4*, were significantly increased during osteogenesis and adipogenesis, respectively (Fig. 1c, d). Therefore, human BMSCs displayed typical adipogenic and osteogenic lineage characteristics after oriented induction.

### Advantages of multi-strategic RNA-seq analysis of induced BMSCs

**Fig. 2:**
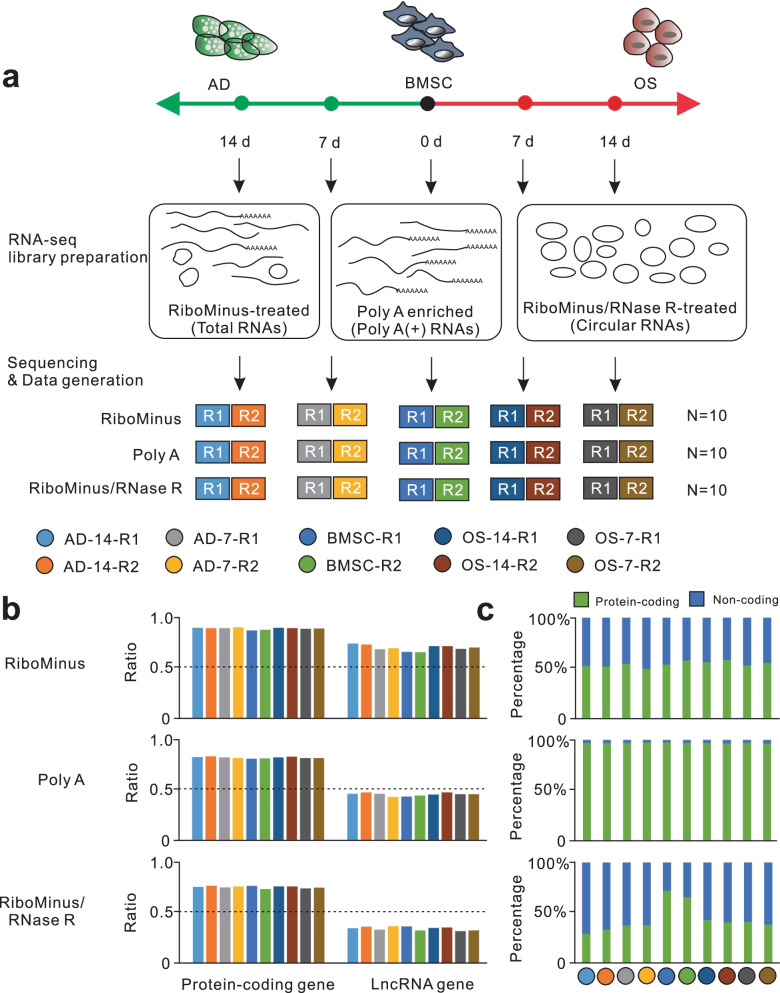
Overview of the multi-strategic RNA-seq of BMSCs. **a** Experimental design of RNA-seq. The RiboMinus, Poly A and RiboMinus/RNase R-treated libraries were constructed to detect total, Poly A(+) and circular RNAs, respectively, at five time point of adipo-osteogenesis of BMSCs with two replicates for each type of library and time point. **b** The ratios of annotated genes detected from each RNA-seq data. **c** The comparison percentages of protein-coding and non-coding genes detected in each RNA-seq data

To generate the comprehensive RNA landscape of induced BMSCs, we sequenced 30 samples from five time points during adipo-osteogenesis of human BMSCs, using three RNA-seq library types: (1) RiboMinus-treated library for total RNA detection, (2) Poly(A) enrichment library as standard RNA-seq library, (3) RiboMinus/RNase R-treated library for circRNA detection (10 libraries for each type) (Fig. 2a; Additional file 2: Table S1). For the three types of RNA-seq libraries, an average of 62 million clean reads were obtained, 94% of which were mapped to the human genome (version hg38) (Additional file 1: Fig S2a; Additional file 2: Table S1). The expression profiles of both protein-coding and lncRNA genes of two replicates from each library type and time point exhibited high correlations, indicating excellent repeatability of our experiments (Additional file 1: Fig S2b, c). The expressional changes of adipo-osteogenic marker genes showed similar trends when detected by RNA-seq or qPCR (Fig. 1c; Additional file 1: Fig S2d). Furthermore, we found that an average of 87% of protein-coding genes and 68% of lncRNA genes annotated by the GENCODE database were detected in RiboMinus data, whereas these proportions were 80% and 44% in Poly A data, 74% and 34% in RiboMinus/RNase R data (Fig. 2b). An average of 97% of the detected genes in Poly A data belonged to protein-coding genes, whereas the proportions were 54% in RiboMinus data, and 47% in RiboMinus/RNase R data (Fig. 2c). In addition, the highest portions of back-spliced junction (BSJ) reads were detected in RiboMinus/RNase R data (Additional file 1: Fig S2e; Additional file 2: Table S1). Therefore, both RiboMinus- and RiboMinus/RNase R-treated RNA-seq methods preserved the balanced RNA proportions of protein-coding and lncRNA genes, while the RiboMinus method detected slightly more annotated genes and the RiboMinus/RNase R method detected extraordinarily more BSJ reads, which is valuable for circRNA identification. The Poly A data displayed a strong bias for the enrichment of RNAs from protein-coding genes. These results demonstrated that the three library types exhibited distinct advantages in providing an overall RNA landscape during adipo-osteogenesis of human BMSCs.

### Integrative identification of circRNAs in inducted BMSCs

**Fig. 3:**
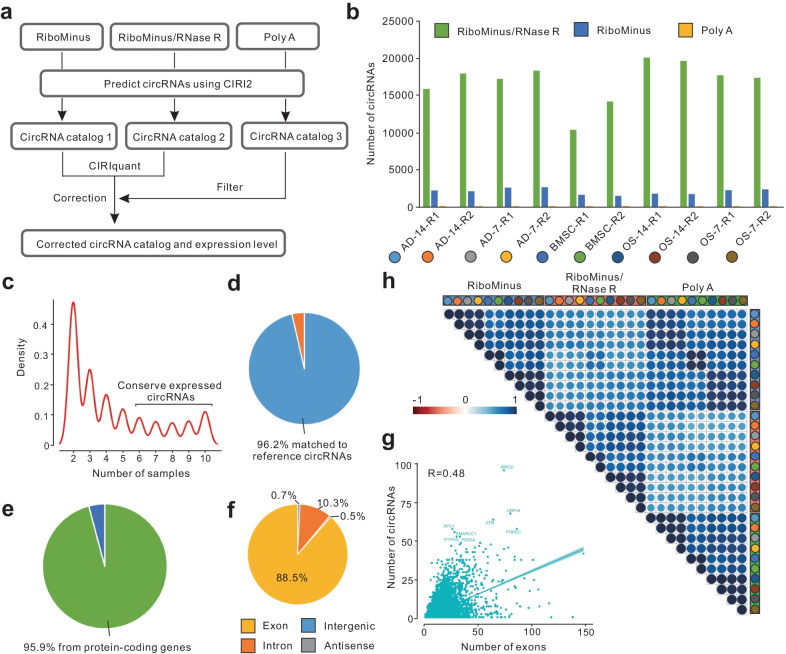
Integrative identification of circRNAs of adipo-osteogenesis of BMSCs.** a** Flowchart of the overall approach. The circRNAs were predicated from each multi-strategic sequencing data, filtered with false-positive signals using Poly A data, and corrected for RNase R efficiency using RiboMinus and RiboMinus/RNase R data. **b** Numbers of identified circRNAs from different sequencing libraries. **c** Distribution of identified circRNAs for the numbers of samples. **d** Percentage of determined circRNAs with annotated reference. **e** Percentage of identified circRNAs derived from protein-coding genes. **f** Distribution of identified circRNAs in genomic regions. **g** Correlation analysis between the numbers of gene exons and the numbers of derived circRNAs. **h** Correlation matrix of all samples for genes with the potential to generate circRNAs

To improve the accuracy of circRNA identification, an integrative pipeline was performed to utilize the advantages of multiple dataset types. The predicted circRNAs were detected using the CIRI2 tool for each library type, while predicted circRNAs from Poly A-treated libraries were considered as false-positive signals and filtered out. The expression values of the remaining circRNAs in the RiboMinus and RiboMinus/RNase R data were corrected for RNase R efficiency using the CIRIquant tool (Fig. 3a). According to the circRNA identification results, at least eight times more circRNAs were identified in each RiboMinus/RNase R data set (16,767 circRNAs) than those in RiboMinus data (2,063 circRNAs), while an average of 14 circRNAs were identified and filtered from the Poly A data (Fig. 3b). After correction and merging, a total of 48,152 circRNAs were obtained from 7366 genes (Additional file 3: Table S2), of which 30% were identified in more than five samples and thus may be considered conserved circRNAs (Fig. 3c). Among these circRNAs, 96.2% were matched with circAtlas 2.0 annotation, 95.9% were generated from protein-coding genes, and 88.5% were from exonic regions (Fig. 3d–f).

Next, we evaluated the relationship between host gene characteristics and their circRNA numbers. The results showed that there was a higher correlation between the number of host gene exons and the number of their circRNAs than between the host gene expression levels and the numbers of their circRNAs (Fig. 3g; Additional file 1: Fig. S2f). For example, BIRC6 has 74 exons and generated 96 circRNAs. Furthermore, the circRNA correlation analysis, based on the expression levels of 7,366 host genes, showed a high correlation between two replicates at each time point from each type of library and among different lineages. This suggested that circRNA-related genes exhibited lineage-specific expression characteristics (Fig. 3h).

### Lineage-specific expression of circRNA genes during adipo-osteogenesis of BMSCs

**Fig. 4:**
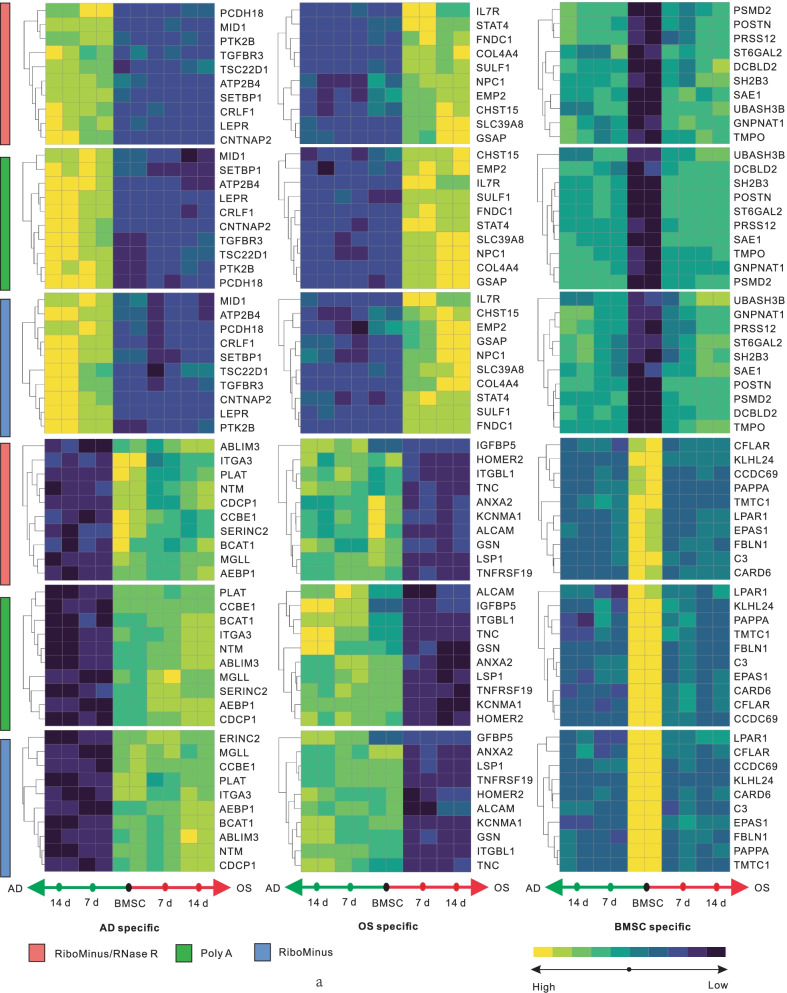
Dynamic expression profiles during adipo-osteogenesis of BMSCs. The significantly changed host genes of circRNAs (adjusted *P* value < 0.05 & |log2 FoldChange|> 1) were analyzed in RiboMinus/RNase R data. Top ten significant ones in different lineages were presented in the heatmaps, along with their related expression patterns in Poly A and RiboMinus data. Yellow indicated high expression levels, and blue indicated low expression levels

The efficient differentiation of MSCs requires genome-wide gene-specific expression at different developmental stages. To characterize the distinct patterns of circRNA expression more comprehensively, we performed differential gene expression analysis, using the circRNAs we identified as a reference, during adipo-osteogenesis of BMSCs. In total, 2,077 circRNA genes were found to be significantly differentially expressed (adjusted *P* value < 0.05 & |log2 FoldChange|> 1) and were further grouped into three distinct expression patterns (Additional file 4: Table S3). Especially, 969 circRNA genes were significantly up- or down-regulated during adipogenic process and were classified as pattern I (AD-specific), represented by *PTK2B* [33], *TGFBR3* [34], *LEPR* [35] and *AEBP1* [36], whose host genes were key regulators of adipogenesis (Additional file 4: Table S3; Fig. 4). On the other hand, 635 circRNA genes with significantly different expression levels during osteogenesis were classified as pattern II (OS-specific), represented by *COL4A* [37], *SULF1* [38], *SLC39A8* [39], *IGFBP5* [40], *HOMER2* [41] and *ALCAM* [42], whose host genes were involved in osteogenesis (Additional file 4: Table S3; Fig. 4). Besides, 1173 significantly changed circRNA genes in BMSCs were classified as pattern III (BMSC-specific), represented by *LPAR1* [43] and *EPAS1* [44], whose host genes were related to the maintenance of MSC homeostasis (Additional file 4: Table S3; Fig. 4). For those representative circRNA genes detected by RiboMinus/RNase R method, their corresponding RNAs detected by RiboMinus and Poly A methods showed similar expressional trends (Fig. 4). Taken together, the results suggested that the circRNAs identified for each pattern may be orchestrated and served as the functional program to regulate MSC differentiation.

### Comparison analysis of circRNAs between induced BMSCs and AMSCs

**Fig. 5:**
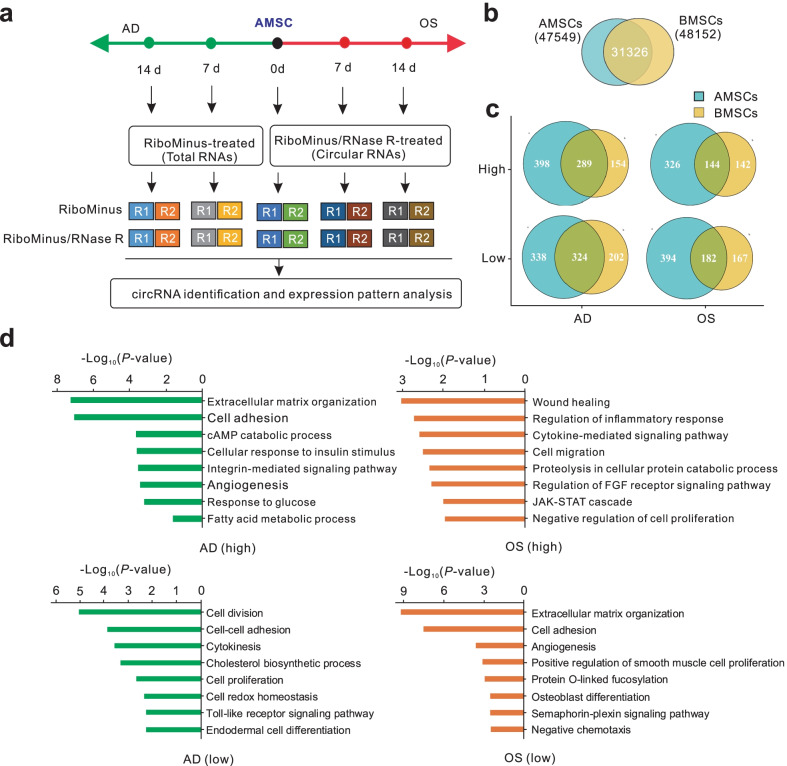
Comparisons of circRNAs identified from AMSCs and BMSCs. **a** Flowchart for identifying circRNAs in differentiated AMSCs. **b** Overlap of identified circRNAs from induced AMSCs and BMSCs. **c** Overlap of lineage-specific host genes of circRNAs from induced AMSCs and BMSCs (adjusted *P*-value < 0.05 & |log2 FoldChange|> 1). **d** Top eight significantly enriched GO terms from host genes of adipogenesis- or osteogenesis-specific circRNAs

AMSCs are important source for stem cell therapies in addition to BMSCs [45, 46]. The universal clinical application of MSCs from diverse sources requires an understanding of their common mechanisms. Therefore, we established an adipo-osteogenesis system of AMSCs using the same induction medium as the medium used for BMSCs (Additional file 1: Fig. S3). Subsequently, we sequenced 20 samples from differentiated AMSCs at five time points using RiboMinus- and RiboMinus/RNase R-treated methods. We generated an average of 77 million clean reads, of which 92% were precisely mapped to the human genome. BSJ reads were highly abundant in RiboMinus/RNase R data (Fig. 5a; Additional file 1: Fig. S4; Additional file 2: Table S1). After a comparison analysis, 31,326 circRNAs were collectively identified in AMSC- and BMSC-derived datasets, among which 1,166 circRNA genes were significantly changed among different lineages and grouped into AD-specific (613 genes), OS-specific (326 genes) and MSC-specific patterns (168 genes) (Fig. 5b; Additional file 4: Table S3; Additional file 5: Table S4). Gene Ontology functional analysis showed that the host genes in AD-specific pattern were related to cell adhesion [47], glucose [48, 49] and lipid metabolism [50, 51], which are crucial for adipogenesis. The host genes in OS-specific pattern were involved in cytokine signaling [52], cell migration [53] and growth factor signaling [54], which are important in the regulation of bone formation. The host genes in the MSC-specific pattern were mainly related to cell division related processes [55], which are important for controlling the pluripotency and differentiation of stem cells (Fig. 5d; Additional file 6: Table S5). Thus, a considerable number of lineage-specific circRNA genes were shared by MSCs from both bone marrow and adipose, and their host genes were involved in distinct functional sets related to differentiation and stemness, which provided the potential common regulators of adipo-osteogenesis processes.

### Validation of circRNAs during adipo-osteogenesis of MSCs

**Fig. 6:**
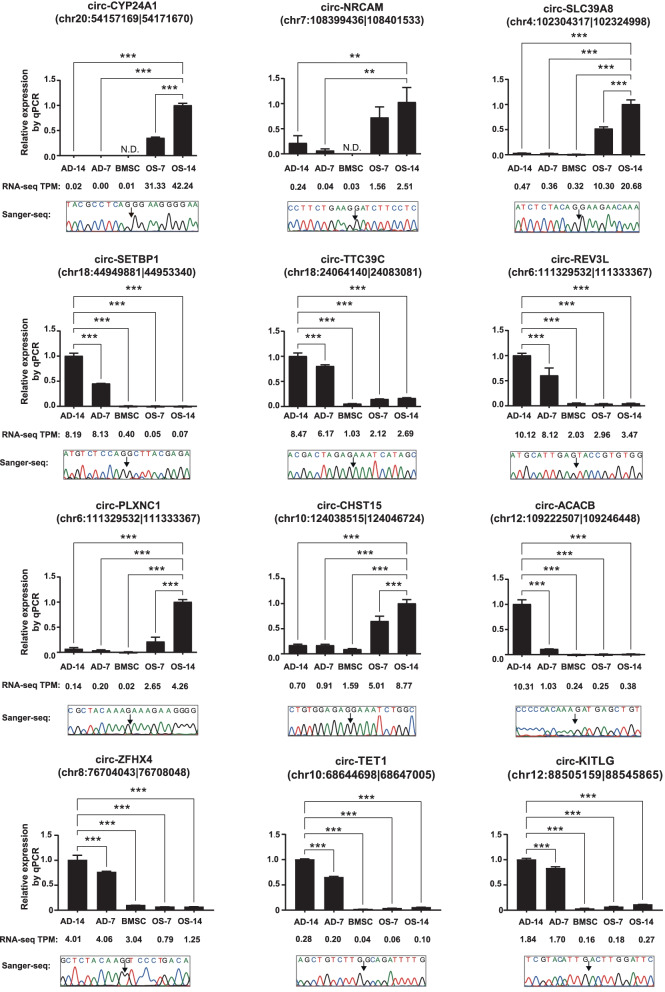
Confirmation of circRNAs by qPCR and Sanger sequencing during adipo-osteogenesis of human BMSCs. The average TPM values of host genes of circRNAs from RiboMinus/RNase R RNA-seq were listed below. Statistically significant differences of circRNAs between multiple groups verse the highest expressed group were performed with Bonferroni’s multiple comparisons test after one-way ANOVA test. All data are presented as means ± SD (*n * = 3). **P < 0.01, ***P < 0.001

To validate the integrative analysis results of multi-strategic RNA-seq data for human BMSCs and AMSCs, we performed qPCR for twelve lineage-specific circRNAs, including osteogenesis-specific (circ-CYP24A1 and circ-SLC39A8, etc.) and adipogenesis-specific circRNAs (circ-SETBP1 and circ-TTC39C, etc.). Divergent primer pairs were designed to amplify regions that included BSJ sites (Additional file 7: Table S6). The *GAPDH* was used as a reference gene for data normalization. The relative expression results of those twelve circRNAs in induced BMSCs and AMSCs showed similar trends when detected by qPCR or RNA-seq, which indicated that the integrative expression analysis of RNA-seq data was of high quality (Fig. 6; Additional file 1: Fig. S5). Besides, the PCR products were further purified and performed with Sanger sequencing to conform the circRNA sequences. As showed in Fig. 6, the sequencing results precisely matched the BSJ sites of those circRNAs. Therefore, the expression data of circRNA during adipo-osteogenesis of human MSCs were reliable and may be used as a resource for studying the balance between the osteogenesis and adipogenesis of human MSCs.

### Circ-CRLF1 was lineage-specially expressed and regulated adipogenesis

**Fig. 7:**
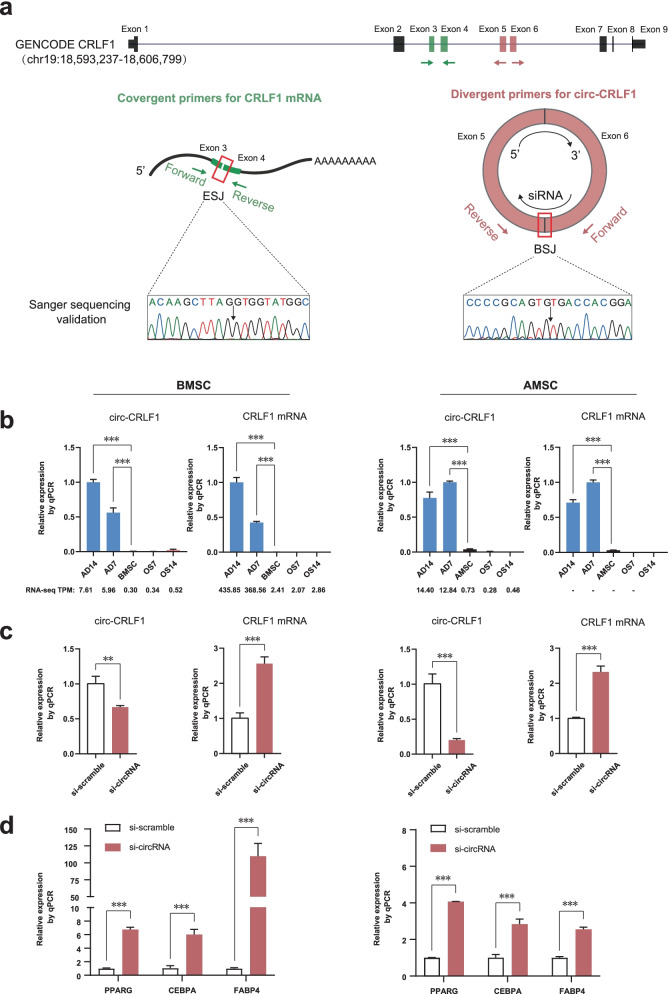
Circ-CRLF1 characteristics during adipo-osteogenesis of MSCs. **a** The primers for circ-CRLF1 and related mRNA were validated by Sanger sequencing. **b** Relative expression of circ-CRLF1 and related mRNA by qPCR. **c** The knock-down efficiency of siRNA for circ-CRLF1 was validated by qPCR. **d** The mRNA expression levels of the markers *PPARG*, *CEBPA* and *FABP4* were elevated at adipogenesis day seven by siRNA. In panel **b**, statistically significant differences of circRNAs between multiple groups verse the highest expressed group were performed with Bonferroni’s multiple comparisons test after one-way ANOVA test. In panel **c** and **d**, statistically significant expressional differences between si-scramble and si-circRNA were determined using Student’s *t*-test. All data are presented as means ± SD (*n* = 3). **P < 0.01, ***P < 0.001

To elucidate the roles of lineage-specific circRNAs in adipo-osteogenesis of MSCs, we performed functional validation of the circRNA gene, *CRLF1*, one of the top ten significantly high-expressed adipogenesis-specific circRNA genes (Fig. 4). Circ-CRLF1 (position, chr19:18596622–18597049; spliced length, 327 nt) was originated from the fifth and sixth exons of the *CRLF1* gene (Fig. 7a). The expression levels of *CRLF1* mRNA decreased sharply after RNase R treatment, while RNase R failed to degrade circ-CRLF1 (Additional file 1: Fig. S6), which confirmed the stability of circ-CRLF1. To detect circ-CRLF1 and its related mRNA at the same time without interference, divergent primers for circ-CRLF1 were designed to cover the BSJ of the fifth and sixth exons, while convergent primers were designed to cover the exon-spliced junction (ESJ) between the third and fourth exon. The specificity of those primers was successfully validated by Sanger sequencing (Fig. 7a). The qPCR results indicated that the levels of both circ-CRLF1 and its corresponding mRNA were significantly increased during adipogenesis, but not osteogenesis in human BMSCs and AMSCs, which were similar to the RNA-seq results (Fig. 7b). Furthermore, we designed a siRNA targeting the BSJ site to knock down circ-CRLF1. After 7 days of adipogenesis induction, circ-CRLF1 expression levels were significantly reduced by the siRNA in both BMSCs and AMSCs, compared with its expression levels after treatment with scramble siRNAs. Meanwhile, the targeted siRNA did not reduce the expression levels of *CRLF1* mRNA (Fig. 7c). The knock-down of circ-CRLF1 significantly increased the expression levels of adipogenesis marker genes, including *PPARG*, *CEBPA* and *FABP4*, in human BMSCs and AMSCs after 7 days of adipogenesis induction (Fig. 7d). Taken together, these results indicated that circ-CRLF1 was lineage-specially expressed during adipo-osteogenesis and regulated the adipogenesis process.

## Discussion

Recent studies have indicated that epigenomic regulators, including ncRNAs, are essential for MSC homeostasis [56]. Compared with other ncRNAs, circRNAs are resistant to degradation due to their circular structure [57, 58]. They have become stable biomarkers and long-acting therapeutic targets of various diseases [59, 60]. The genome-wide expressional studies of circRNAs in osteogenesis or adipogenesis, using single-strategic high-throughput analysis, have been accumulating and helpful for elucidation of the functional roles of circRNAs in bone tissues[17–21]. In the present study, we systematically analyzed the adipo-osteogenesis of both BMSCs and AMSCs using multi-strategic methods. We provided an accurate and generally applicable transcriptomic profile of circRNAs to allow further studies of bone development and dysfunction.

In adipo-osteogenic differentiated MSCs, RiboMinus-treated RNA-seq method generated the highest proportion of coding (87%) and non-coding (68%) transcripts, which were consistent with previous results [61]. Nevertheless, this method could only detect limited numbers of circRNAs, due to the low abundance of circRNAs [24, 62]. RNase R treatment could improve the detection of circRNAs by degrading linear RNAs [22]. We detected 16,767 circRNAs per library in differentiated human BMSCs using the RiboMinus/RNase R-treated strategy. This was at least eight times higher than the number of detected circRNAs using the RiboMinus-treated strategy. However, RNase R treatment may generate bias by preferentially preserving highly-expressed circRNAs [23, 24]. The integrative analysis of RiboMinus data and RiboMinus/ RNase R data could correct RNase R efficiency to improve the quantification of circRNAs [24]. The Poly A method removes most circRNAs during the Poly A selection step, and these data are often used as a blank background [63]. In the present research, we generated the multi-strategic RNA data from RiboMinus, RiboMinus/RNase R, and Poly A libraries for differentiated human BMSCs, and performed integrative analysis using CIRI2 and CIRIquant tools. At last, 48,152 circRNAs from 7366 host genes were identified after systemic correction, and 96.2% of them were overlapped with reference circRNAs, which indicated the high reliability of our data. Similar to previous studies [64], most of the circRNAs that we identified in differentiated human BMSCs were from protein-coding genes and their exons. Thus, our integrative data provided high-quality circRNA annotations for differentiated human BMSCs.

Public RNA-seq datasets have suggested that circRNAs are dynamically expressed in spatial- and temporal-specific manners, and may have essential roles in developmental processes [62, 65]. Therefore, it is crucial to characterize the expression patterns of circRNAs for studies of bone formation and homeostasis. In the present study, we identified 969 adipogenic, 635 osteogenic and 1,173 BMSC-specific host genes of circRNAs. The top ten up- or down-regulated lineage-specific circRNAs shared similar expressional trends with their corresponding total and linear RNAs. Although the correlation between circRNAs and their related linear RNAs have differed from various studies, this may be due to the different selected clusters of circRNAs [66, 67]. Moreover, several host genes of those circRNAs with differential expression levels were involved in lineage-specific activities [33–44]. Thus, these lineage-specific circRNAs may provide regulatory candidates of adipo-osteogenesis of human BMSCs.

MSCs are heterogenous populations and differ depending on their tissue origin or the conditions of the donors (e.g., age, diseases, or unknown factors) [68]. Therefore, it is urgent to identify the common regulatory factors of MSCs for their universal application. In the present study, we annotated 31,326 shared circRNAs and identified 613 adipogenic and 326 osteogenic host genes with significantly altered expression levels in both BMSCs and AMSCs. The shared up-regulated host genes in each lineage of MSCs were related to distinct biological processes, including cell adhesion, cytokine signaling, and cell division, which indicated their potential involvement in the regulation of adipo-osteogenesis of MSCs. In particular, the circRNA of CRLF1 was significantly up-regulated during the adipogenesis of both BMSCs and AMSCs. The knock-down of circ-CRLF1 significantly promoted adipogenesis, which suggested that circ-CRLF1 could be a potential target for the prevention or treatment of bone-related diseases induced by an imbalance of adipo-osteogenesis.

## Conclusion

In summary, we have annotated over ten thousand of circRNAs, and identified various circRNAs that were lineage-specifically regulated during osteogenesis and adipogenesis of human BMSCs and AMSCs. Their host genes were involved in distinct biological processes, including cell adhesion, cytokine signaling, and cell division. Furthermore, we identified a lineage-specific circRNA, circ-CRLF, that negatively regulates adipogenesis. This study paves the way for further investigation to understand the potential roles of circRNAs in bone development and identify the therapeutic targets of bone diseases.

## Supplementary Information

**Additional file 1**. **Figure S1**: Statistical analysis of human BMSCs. **Figure S2**. General features of multi-strategic libraries for adipo-osteogenesis of BMSCs. **Figure S3**. Adipogenesis and osteogenesis of human AMSCs. **Figure S4**. General features of multi-strategic libraries for adipo-osteogenesis AMSCs. **Figure S5**. Confirmation of circRNAs by qPCR and Sanger sequencing during adipo-osteogenesis of human AMSCs. **Figure S6**. The abundant of circ-CRLF1 and CRLF1 mRNA at day 14 during adipogenesis treated with RNase R.

**Additional file 2**. **Table S1**: Detailed information for multi-strategic RNA-seq of adipo-osteogenesis of MSCs.

**Additional file 3**.** Table S2**: Identified circRNAs through integrative analysis of adipo-osteogenesis of BMSCs.

**Additional file 4**.** Table S3**: Significantly differentially expressed circRNA genes in induced BMSCs from RiboMinus/RNase R data.

**Additional file 5**.** Table S4**: Significantly differentially expressed circRNA genes in induced AMSCs from RiboMinus/RNase R data.

**Additional file 6**.** Table S5**: Top ten significantly enriched GO terms for lineage-specific host genes of circRNAs.

**Additional file 7**.** Table S6**: Oligonucleotides used in this study.

## Data Availability

All the datasets supporting the results have been listed in the article and its additional files. The RNA-seq data have been deposited to the National Center for Biotechnology Information (NCBI) Short Read Archive (SRA) under accession code PRJNA688535.

## Abbreviations

MSC: Mesenchymal stem cells
BMSC: Bone marrow-derived mesenchymal stem cells
AMSC: Adipose-derived mesenchymal stem cells
OS: Osteogenesis
AD: Adipogenesis
ncRNAs: Non-coding RNAs
circRNAs: Circular RNAs
RiboMinus: Ribosome RNAs-depleted
RiboMinus/RNase R: Ribosome RNAs-depleted and RNase R-treated
ESJ: Exon-spliced junction
BSJ: Back-spliced junction
