# Supplementary material for: Integrative analysis reveals a lineage-specific circular RNA landscape for adipo-osteogenesis of human mesenchymal stem cells
Source: Stem Cell Res Ther. 2022 Mar 12;13:106. doi: 10.1186/s13287-022-02792-5 (PMC8917624; doi:10.1186/s13287-022-02792-5)
Supplement: Supplementary file 1 — Additional file 1. Figure S1: Statistical analysis of human BMSCs. Figure S2. General features of multi-strategic libraries for adipo-osteogenesis of BMSCs. Figure S3. Adipogenesis and osteogenesis of human AMSCs. Figure S4. General features of multi-strategic libraries for adipo-osteogenesis AMSCs. Figure S5. Confirmation of circRNAs by qPCR and Sanger sequencing during adipo-osteogenesis of human AMSCs. Figure S6. The abundant of circ-CRLF1 and CRLF1 mRNA at day 14 during adipogenesis treated with RNase R. [file 13287_2022_2792_MOESM1_ESM.docx]

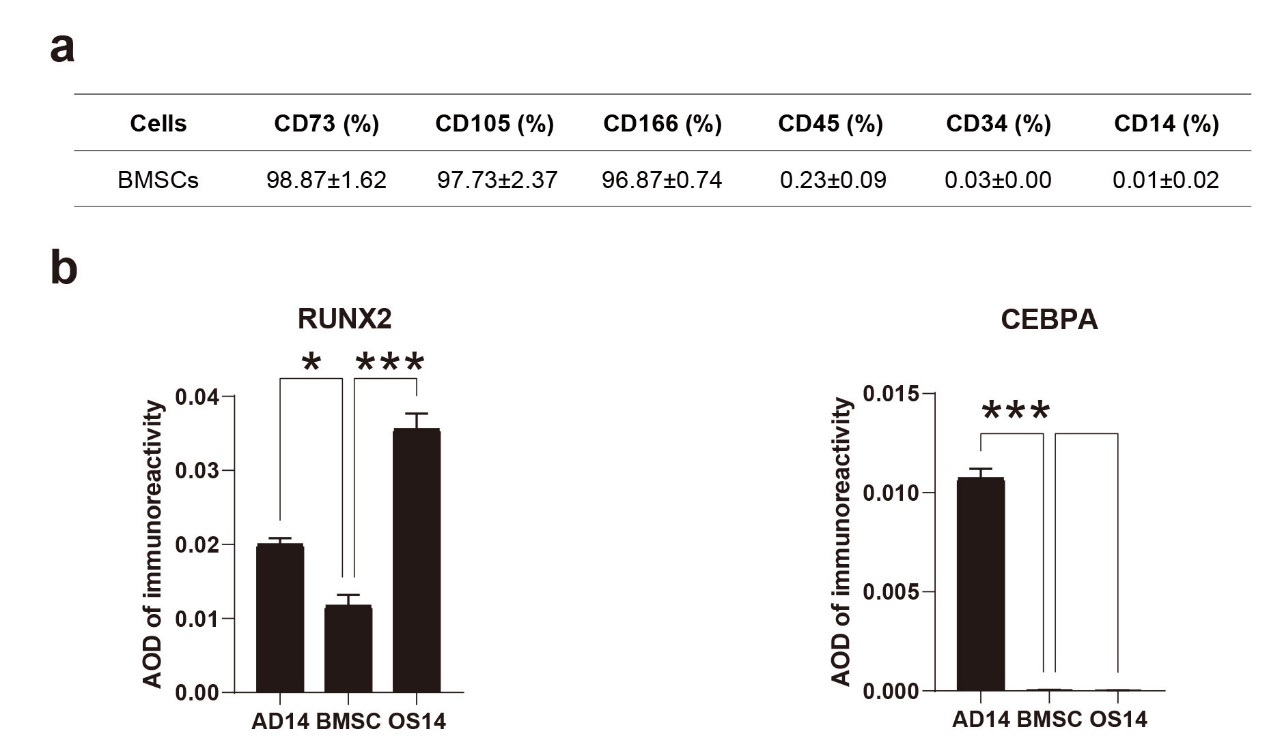


**Additional file: Figure S1** Statistical analysis of human BMSCs. **a** Percentage of populations positive for cell surface markers. **b** AOD of RUNX2 and CEBPA proteins for adipo-osteogenesis at different time points. Statistically significant differences of genes between multiple time points versus day 0 (BMSC) were performed with Bonferroni’s multiple comparisons test after one-way ANOVA test. Scale bars = 200 μm. All data are presented as means ± SD (*n* = 3). **P* < 0.05, ****P* < 0.001.


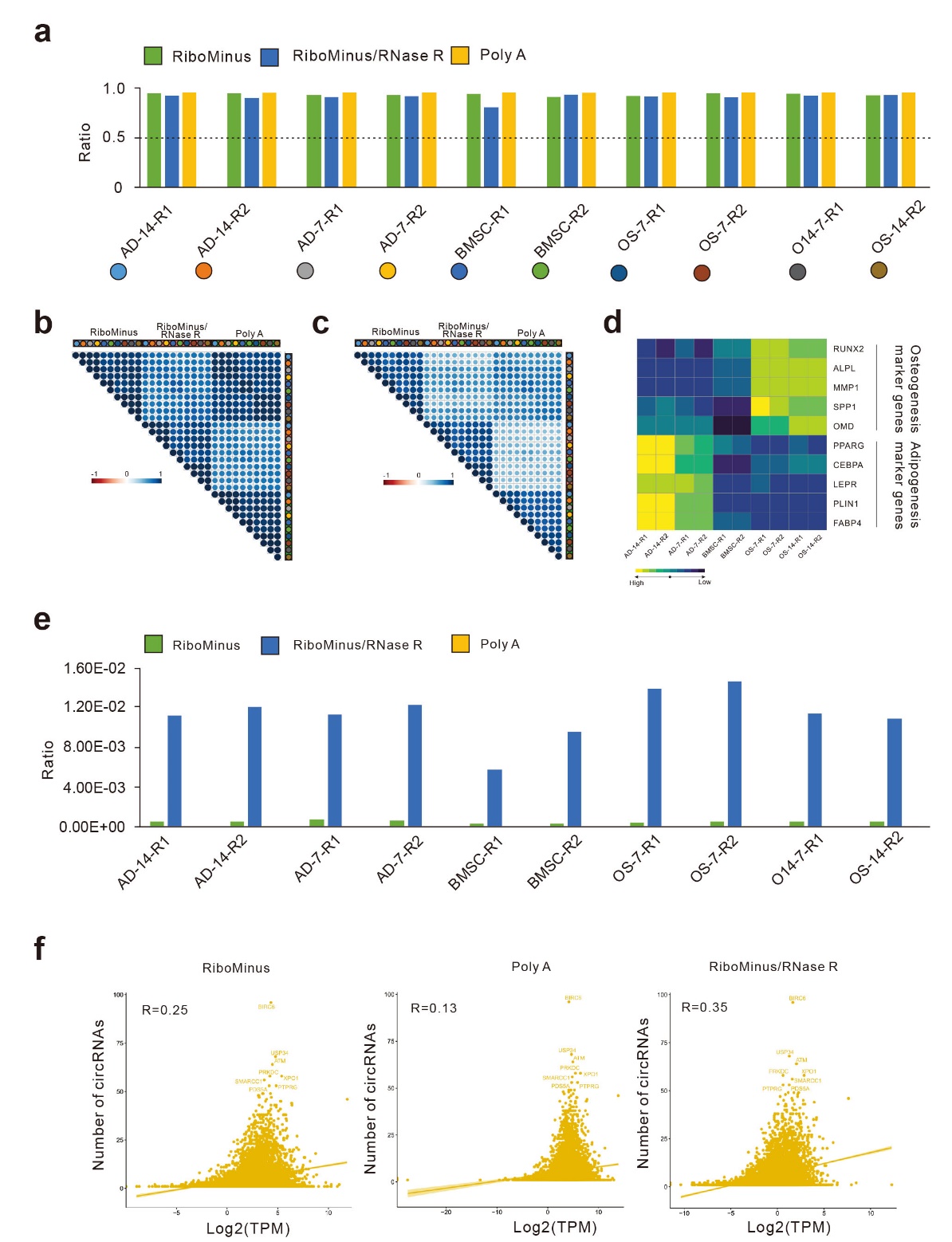


**Additional file: Figure S2** General features of multi-strategic libraries for adipo-osteogenesis of BMSCs. **a** The ratios of reads mapped to the human genome from different RNA-seq. **b-c** Correlation matrix of all 30 RNA-seq samples (based on Pearson correlation coefficients) for protein-coding genes (**b**) and lncRNA genes (**c**). **d** Heatmap of marker gene expressions in Poly A RNA-seq data. **e** Ratios of BSJ reads from different sequencing libraries. **f** Correlation analysis between the expression levels of host genes and the numbers of their derived circRNAs.


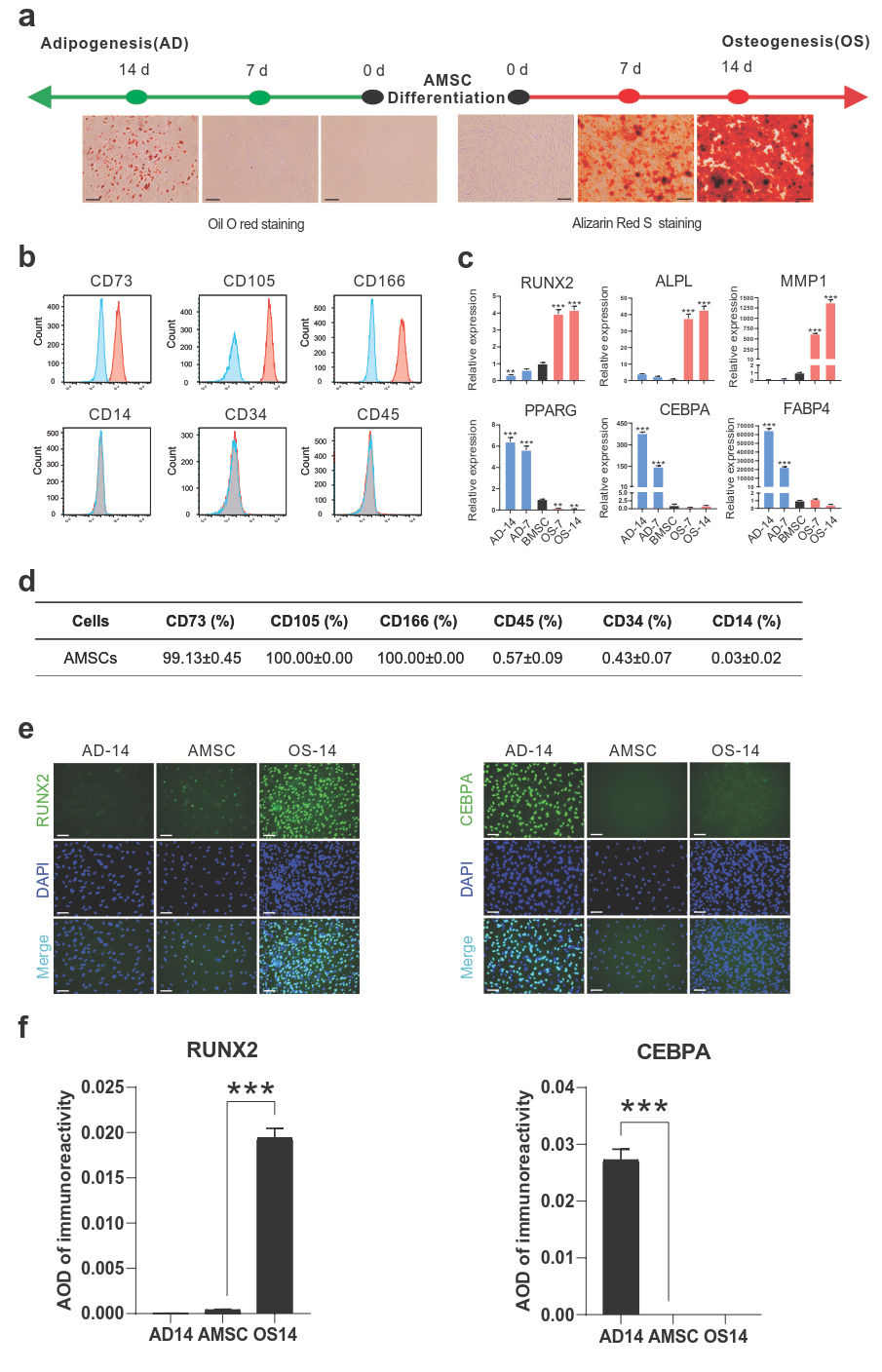


**Additional file: Figure S3** Adipogenesis and osteogenesis of human AMSCs. **a** Oil Red O and Alizarin Red S staining for adipogenic and osteogenic differentiated AMSCs, respectively, on day 0, 7 and 14. **b** Distribution of surface markers of AMSCs. **c** Expression of marker genes. **d** Percentage of populations positive for cell surface markers. **e** Immunofluorescence staining of marker proteins. **f** AOD of RUNX2 and CEBPA proteins for adipo-osteogenesis at different time points. Statistically significant differences of genes between multiple time points versus day 0 (AMSCs) were performed with Bonferroni’s multiple comparisons test after one-way ANOVA test. Scale bars = 200 μm in pannel **a**, scale bars = 75 μm in pannel **e**. All data are presented as means ± SD (*n* = 3). ***P* < 0.01, ****P* < 0.001.


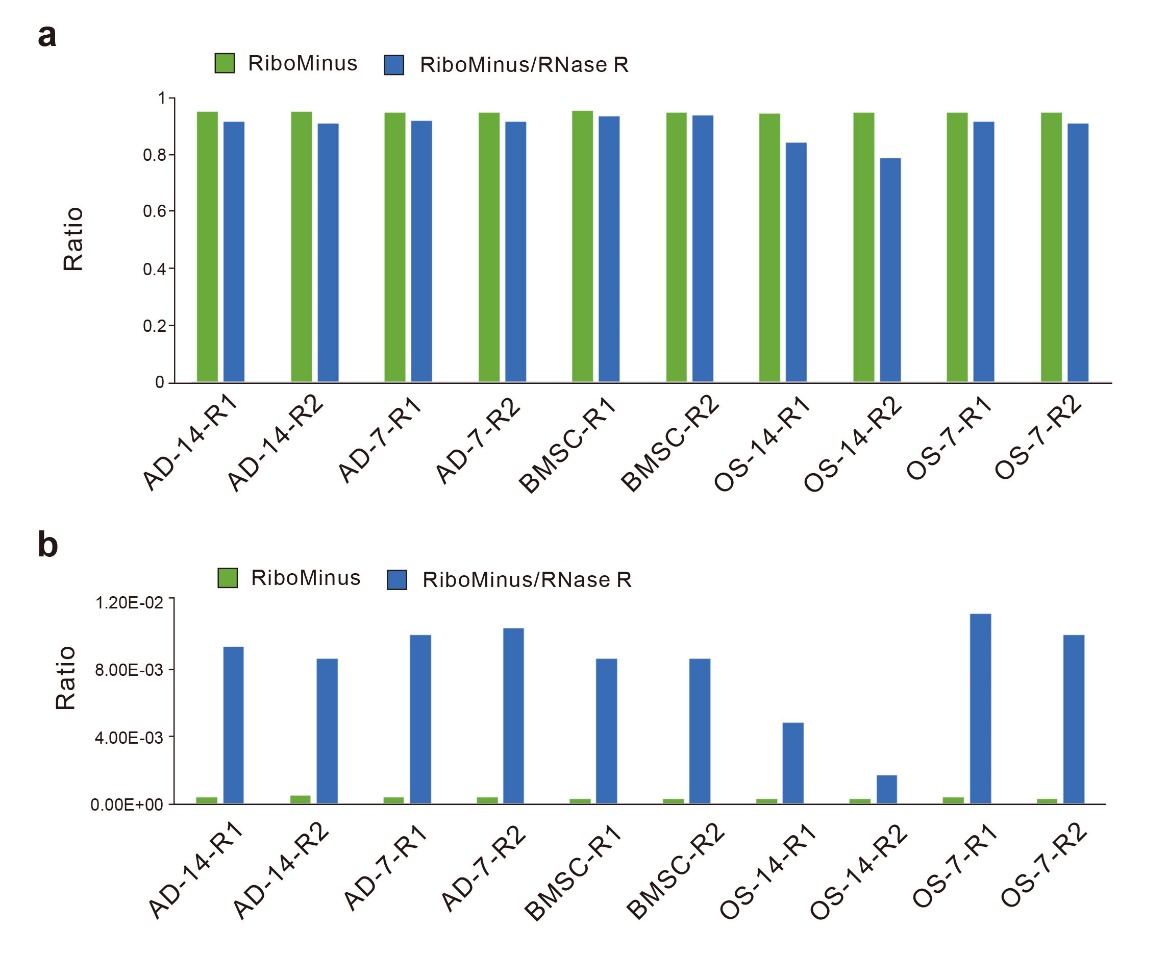


**Additional file: Figure S4** General features of multi-strategic libraries for adipo-osteogenesis AMSCs. **a** The ratios of reads mapped to the human genome from different RNA-seq. **b** Ratios of BSJ reads from different RNA-seq.


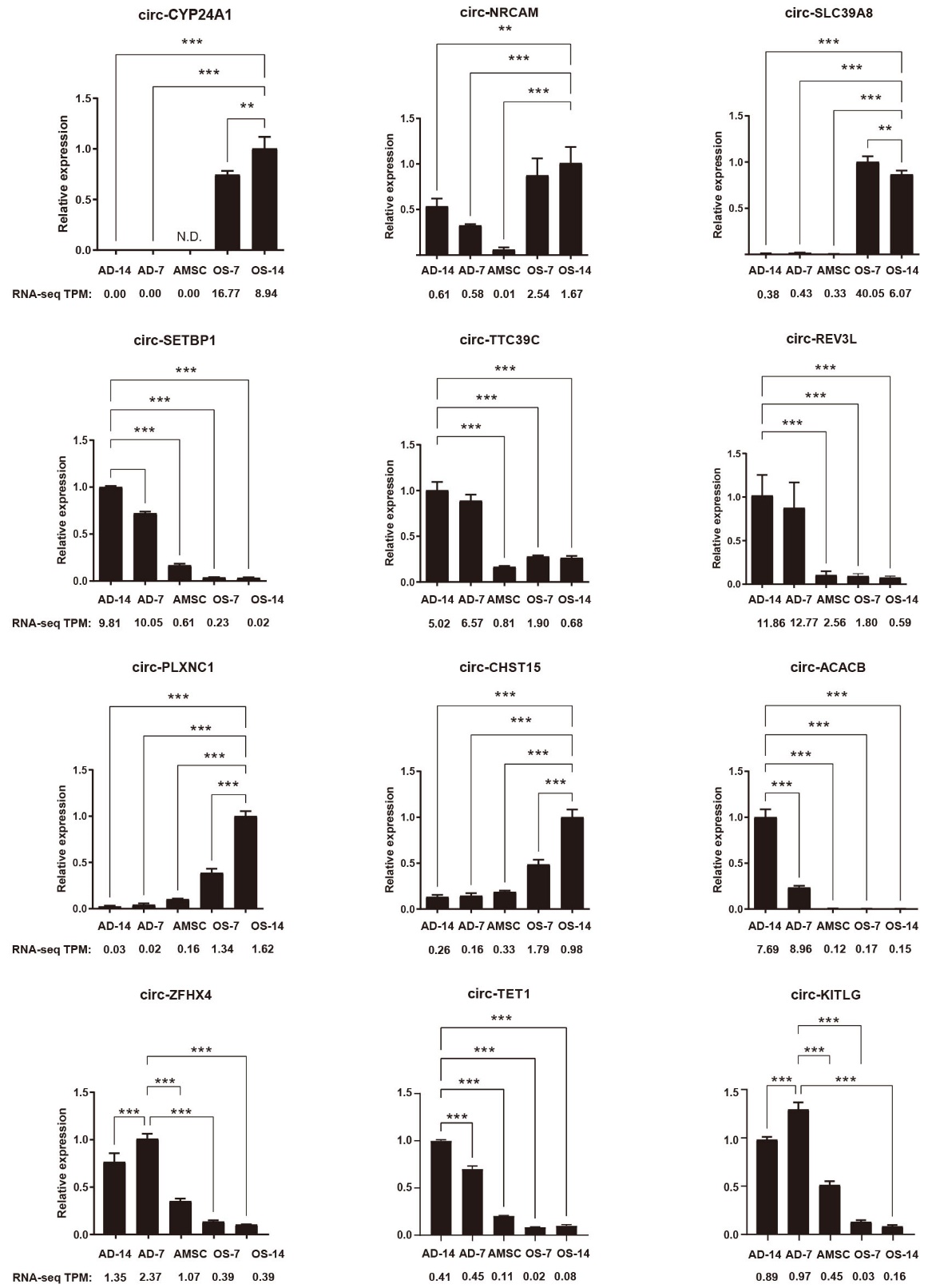


**Additional file:** **Figure S5** Confirmation of circRNAs by qPCR and sanger sequencing during adipo-osteogenesis of human AMSCs. The average TPM values of host genes of circRNAs from RiboMinus/RNase R RNA-seq were listed below. Statistically significant differences of circRNAs between multiple groups verse the highest expressed group were performed with Bonferroni’s multiple comparisons test after one-way ANOVA test. All data are presented as means ± SD (n = 3). ***P* < 0.01, ****P* < 0.001.


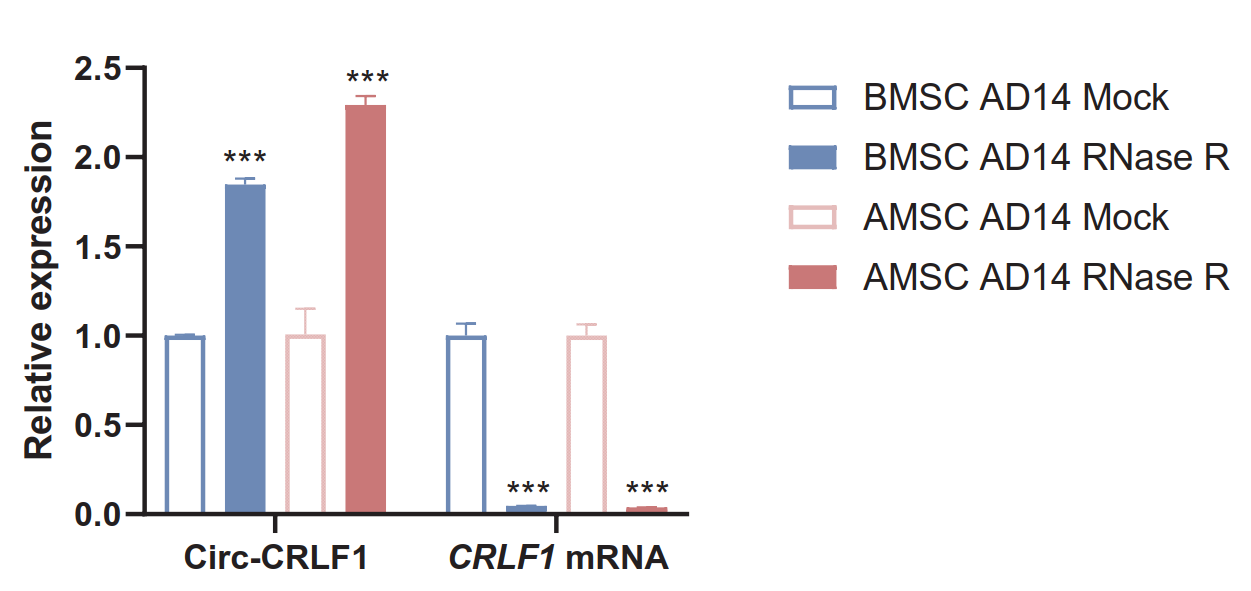


**Additional file: Figure S6** The abundant of circ-CRLF1 and *CRLF1* mRNA at day 14 during adipogenesis treated with RNase R. Statistically significant differences of RNAs between RNase R treatment group and mock group were compared using a two-tailed Student’s *t*-test. All data are presented as means ± SD (n = 3). ****P* < 0.001.

s
